# Lyme Disease Transmission Risk: Seasonal Variation in the Built Environment

**DOI:** 10.3390/healthcare6030084

**Published:** 2018-07-19

**Authors:** Amanda Roome, Rita Spathis, Leah Hill, John M. Darcy, Ralph M. Garruto

**Affiliations:** 1 Department of Anthropology, Binghamton University, Binghamton, NY 13902, USA; aroome1@binghamton.edu; 2 School of Pharmacy and Pharmaceutical Sciences, Binghamton University, Binghamton, NY 13902, USA; rspathis@binghamton.edu; 3 Quality Control, Regeneron Pharmaceuticals, Albany, NY 12144, USA; lhill5@binghamton.edu; 4 US Clinical Development & Medical Affairs in the Division of Immunology, Hepatology and Dermatology, Novartis, East Hanover, NJ 07936, USA; jdarcyi1@binghamton.edu; 5 Department of Biological Sciences, Binghamton University, Binghamton, NY 13902, USA

**Keywords:** tick-borne diseases, *Borrelia burgdorferi*, tick density and infection rate, human risk factors, Northeastern United States

## Abstract

Seasonal variation in spatial distribution and pathogen prevalence of *Borrelia burgdorferi* in blacklegged ticks (*Ixodes scapularis*) influences human population risk of Lyme disease in peri-urban built environments. Parks, gardens, playgrounds, school campuses and neighborhoods represent a significant risk for Lyme disease transmission. From June 2012 through May 2014, ticks were collected using 1 m^2^ corduroy cloths dragged over low-lying vegetation parallel to walkways with high human foot traffic. DNA was extracted from ticks, purified and presence of *B. burgdorferi* assessed by polymerase chain reaction amplification. Summer is reported as the time of highest risk for Lyme disease transmission in the United States and our results indicate a higher tick density of 26.0/1000 m^2^ in summer vs. 0.2/1000 m^2^ to 10.5/1000 m^2^ in spring and fall. However, our findings suggest that tick infection rate is proportionally higher during the fall and spring than summer (30.0–54.7% in fall and 36.8–65.6% in spring vs. 20.0–28.2% in summer). Seasonal variation in infected tick density has significant implications for Lyme disease transmission as people are less likely to be aware of ticks in built environments, and unaware of increased infection in ticks in spring and fall. These factors may lead to more tick bites resulting in Lyme infection.

## 1. Introduction

Emerging infectious diseases (EID’s) and re-emerging infectious diseases are a significant and growing problem affecting population health and place an increasingly heavy burden on public health infrastructure globally by stressing individuals, families and communities [1,2,3,4,5]. While many factors contribute to EID’s, the intersection of ecological and environmental factors with human behavioral patterns are increasingly recognized as fundamental to the transmission of zoonotic diseases [6,7,8,9]. Currently, zoonoses represent the majority of EID’s in humans [3,10,11], with vector-driven zoonoses emerging due to societal, demographic and climatic changes [12,13,14,15].

Lyme disease, caused by *Borrelia burgdorferi* sensu lato complex, a spirochetal bacterium, is the most common vector-borne disease in the United States and is transmitted to humans via the blacklegged tick, *Ixodes scapularis* (formerly known as the deer tick), in the Northeast and Upper Midwest, and transmitted by *Ixodes pacificus* on the West Coast [16,17,18,19]. The ensuing multi-systemic bacterial infection can result in flu-like symptoms, fever, fatigue, joint pain, musculoskeletal pain, headaches, sleep disturbances and depression, among other symptoms [20,21,22,23]. The disease is also known for erythema migrans (EM), a rash that sometimes resembles a “bull’s eye”; however, EM is not associated with every case of Lyme, and can also manifest in a solid, spreading nontarget skin lesion [24,25,26]. Untreated, the disease can result in serious neurologic and cardiac complications (potentially manifesting as myocarditis, pericarditis, pancarditis, dilated cardiomyopathy, and heart failure) [21,27]. Currently, the Centers for Disease Control and Prevention estimates that 300,000 new cases of Lyme disease occur annually, with 95% of reported cases occurring in 14 states in the Northeast and Upper Midwest [28,29,30]. Between 2004 and 2016, tick-borne diseases more than doubled, and were the majority (77%) of all vector-borne diseases reported [31]. The continuing upward trend in cases and its geographic expansion in the United States, Canada and temperate parts of Eurasia make Lyme disease a growing concern for population health in these regions [32,33].

We define built environments, according to the criteria of Srinivasan and colleagues [34], as places where people live, work and spend their leisure time, such as parks, school campuses, neighborhood backyards and other human-made or altered external environmental space where people are regularly perambulating or congregating. These are peri-urban environments in which infectious ticks and the transmission of tick-borne diseases can occur [35,36]. These peri-urban spaces, with fragmented landscapes [32] are conducive to the transmission of zoonotic diseases to human populations and may remain overlooked in terms of Lyme disease risk and management by local communities. Unlike rural or remote hiking, camping, fishing, hunting or other outdoor activities that primarily take place during the summer months, humans within built environments interact year-round, which may leave them at a heightened risk of exposure to infected ticks during spring, summer and fall [35,36,37].

Ecological factors and forest fragmentation are known to have a positive correlation with tick density and infection prevalence of *B. burgdorferi* [37,38]. The initiation and duration of the tick life cycle is an important factor in the overall impact of the environment on tick populations. *Ixodes scapularis*, the blacklegged tick, has a typical life cycle of two years, during which it takes three blood meals, one at each stage of development (larval, nymphal and adult) [39,40]. As *B. burgdorferi* is not transmitted transovarially, larval stage ticks hatch from uninfected eggs [41,42]. It is worth noting, however, that another species of *Borrelia*, *B. miyamotoi,* which is also found in New York, can be transmitted transovarially at a rate of 6–73% [43,44]. Larval ticks take a blood meal during the summer, molt into nymphal ticks, which overwinter and take a blood meal the following spring or summer. Nymphal ticks then molt into adult ticks and take their final blood meal during the fall, with mating typically occurring on a vertebrate host. Females then drop off the host, overwinter and lay eggs the following spring. If, however, females do not mate and feed during the fall, they overwinter, emerge in the spring and take a blood meal, mate and lay eggs [40]. Once infected, a tick, whether in the nymphal or adult stage, is able to transmit the spirochetes causing Lyme disease to other hosts, including humans [45,46].

In New York and the Northeastern United States, many fragmented landscapes within built environments see high human activity during summer months, with a majority of Lyme disease cases reported between May and August [47]. However, human activities continue throughout the year, with significant exposures during the fall and spring months. Larval ticks may become infected after their first blood meal, then molt into nymphs, which are very small and hard to detect. The nymphal stage, which primarily appears between May and August, has been reported to cause most cases of Lyme disease [48,49,50]. Likewise, summer is when large numbers of people typically spend time outdoors, posing an increased risk of Lyme disease transmission [40]. In the present study, we determine the spatial distribution of *I. scapularis* ticks and the prevalence of the Lyme disease pathogen (*B. burgdorferi*) to assess the risk of infection during all seasons of the year, especially in built environments with fragmented landscapes. In the Northeastern United States and Upper Midwest, little data on tick infection rates within built environments across all seasons currently exists, except for the Hudson Valley and Long Island [51,52].

## 2. Materials and Methods 

**Figure 1:**
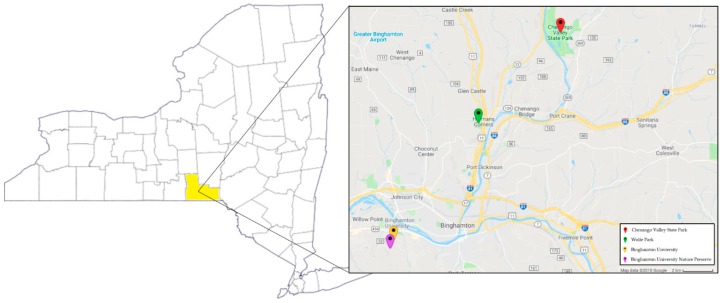
Map of New York State with Broome County (study location) highlighted in yellow. On the right side of the figure are each of the four field sites.

**Figure 2:**
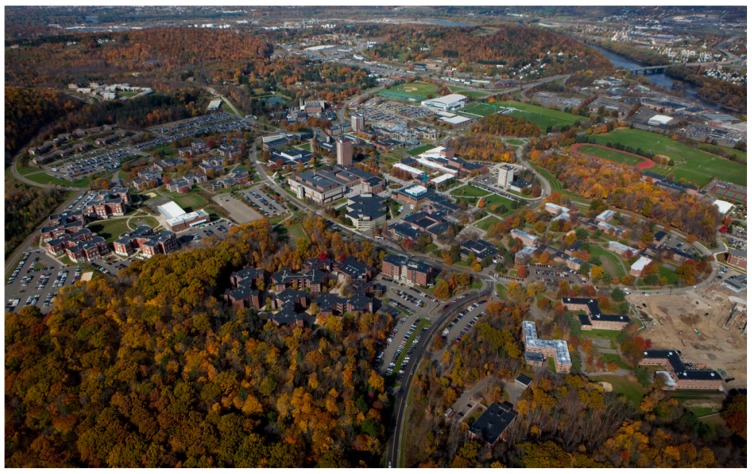
An aerial view of peri urban Broome County, representing a built environment, with interspersed fragmented landscapes and microecologies.

This study was conducted over the course of two years, from June 2012 to May 2014 in the Southern Tier region of upstate New York State along 50 walkways intensively used by humans on the 376.4 hectare Binghamton University campus, its adjacent Nature Preserve (73.7 ha), and in Chenango Valley State Park (460.1 ha) and Wolfe Park (73.7 ha), all within peri-urban Broome County (Figure 1). All sites encompass an assortment of ecological niches that are surrounded by residential, commercial and woodland areas with high human activity (Figure 2). These settings provide ample opportunity for community members, who are interacting with their environments, to come in contact with infected ticks. Seasonality was categorized as follows: spring; April and May, summer; June through August, and fall; September through November. Ticks were not collected December through March due to snow and cold weather and thus low tick activity.

### 2.1. Tick Collection

Along 50 walkways with high human foot traffic on the Binghamton University campus (two sites) and in two parks within Broome County, we designed a specific methodology to assess tick density by collecting ticks three consecutive meters on both sides of and parallel to walkways and paths [53] by dragging a 1 m^2^ white corduroy cloth over low lying vegetation and leaf litter [54,55,56]. Tick collection took place between June 2012 and May 2014, with each walkway being dragged 2–3 times per month. Questing ticks (in search of a blood meal) were removed from the cloth with forceps, placed into sterile cryovials containing 70% ethanol and stored at −20 °C until DNA extraction. 

Density for nymphal and adult ticks was determined by calculating the total area dragged in square meters, either from direct path and walkway measurements, or from existing known path and walkway distances. The number of ticks collected was divided by the total area dragged and multiplied by 1000, resulting in the density of ticks per 1000 m^2^.

### 2.2. Prevalence of B. burgdorferi in Ticks

For each tick species, life cycle stage, sex and collection location were identified. Ticks were then flash frozen in liquid nitrogen and were physically disrupted using chrome steel beads (Biospec Products, Bartlesville, OK, USA) with a TissueLyser LT bead mill (Qiagen, Germantown, MD, USA). DNA was extracted using a Qiagen DNeasy Blood and Tissue Kit according to the manufacturer’s instructions. The presence of *B. burgdorferi* in 1200 ticks was assessed using LD1/LD2 pathogen specific primers targeting the 16S rRNA sequence [57]. For 195 tick samples, *B. burgdorferi* was assessed by *OspC* polymerase chain reaction amplification as a means of determining specific genotypes [58]. When cross checking positivity rates in a sub-sample of 51 ticks between *OspC* and LD1/LD2 primers, infection rates were 98% similar, therefore, there was not a noticeable difference between primer sets.

PCR amplification was performed in 12.5 μL reaction containing 0.5 units HotStar Taq (Qiagen, Germantown, MD, USA), 1.5 mM MgCl_2_, 0.2 mM dNTPs, and 200 nM of each of the primers. The primers sets used were either LD1 and LD2 (LD1: ATGCACACTTGGTGTTAACTA, LD2: GACTTATCACCGGCAGTCTTA [58], or OPSC_4F and OSPC_693R (OPSC_4F: GAAAAAGAATACATTAAGTG, OSPC_693R: GACTTTATTTTTCCAGTTACTTTTTT [59]). Thermal cycling was performed using a GeneAmp^®^ PCR System 9700 (Applied Biosystems, Foster City, CA, USA) with the following program: 15 min at 94 °C, 45 cycles of 94 °C for 15 s, 55 °C for 30 s, and 72 °C for 45 s, followed by 5 min at 72 °C. The presence of PCR product was assessed by agarose gel electrophoresis.

### 2.3. Statistical Analyses

Logistic Regression analyses were conducted using IBM SPSS Statistics Version 19.0 (IBM Corp., Armonk, NY, USA). A Logistic Regression Model was used to determine which variables predicted the outcome of tick infection rates. Z-tests for proportions were used to determine significance between tick infection rates and chi-square tests used to determine significance between overall tick densities.

## 3. Results

Along walkways from four sites in Broome County over the two-year period (June 2012–May 2014), a total of 1375 ticks (481 nymphs and 894 adults) were collected by dragging an area equivalent to 12.7 hectares (126,612.6 m^2^) (Table 1).

### 3.1. Tick Density

Along walkways with high human foot traffic, 1375 ticks were collected, with an overall density of 10.9/1000 m^2^. The highest tick density was found in nymphal ticks during the summer of 2013 at 26.0/1000 m^2^ (Table 1). Summer 2012 density data is not available as walkways and paths were not measured during this initial phase of the study. Adult tick density was slightly higher in fall 2012 at 10.5/1000 m^2^ compared to fall 2013 at 8.6/1000 m^2^.

### 3.2. Tick Infection Rate

Along walkways with high human use, DNA analyses revealed an overall *B. burgdorferi* infection rate of 39.0%, with 27.5% infection rate in nymphal ticks and 45.5% in adult ticks for the time period spanning summer 2012 through summer 2014 (Table 1). Tick infection rates were also calculated by season. The two seasons with the lowest tick infection rates were summer 2012 with a nymphal infection rate of 21.7% and an adult infection rate of 20.0% and summer 2013, with a nymphal infection rate of 28.2%. Only one adult tick was collected during summer of 2013. The highest infection rates were observed in fall 2012, with nymphal and adult infection rates of 50.0% and 54.7%, respectively, and in spring 2014, with an adult infection rate of 65.6% (Table 1). However, the nymphal tick infection rate in fall 2012 was based on only 4 ticks.

### 3.3. Seasonality

**Figure 3:**
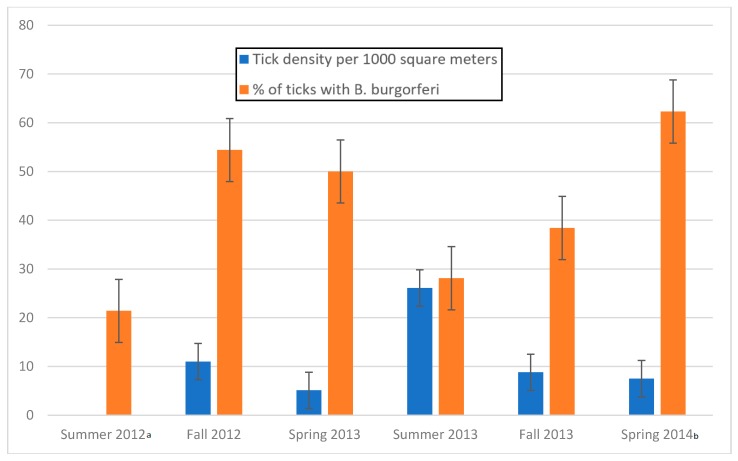
Tick density and infectivity (nymphal and adult) by season during the period Summer 2012 through Spring 2014 with standard error bars. ^a^ Density data for summer 2012 was unavailable, as distances dragged were not determined during that time. ^b^ Spring 2014 infectivity data in this figure represents *OspC* primer sets.

Density of infected ticks was also determined by season (Table 2, Figure 3).

To statistically determine the impact of life cycle stage and season on infection rate, a logistic regression was run. Infection rates in both fall 2013 and spring 2014 were significantly higher than summer 2013 (*p* = 0.02 and *p* = 0.01, respectively). The overall likelihood ratio of the effect of season on the outcome of infection rate was statistically significant at *p* < 0.01.

## 4. Discussion

The rise in incidence of Lyme disease in the Northeastern United States is said to be associated with a myriad of factors, including landscape modification, due in part to suburbanization, climate change, and migratory bird routes [12,15,48,60,61,62,63]. The continued expansion of built environments creates fragmented landscapes where human exposure to infected tick populations is more likely [35,36].

Our study finds that overall tick infection rates in both nymphal and adult ticks with *B. burgdorferi* along walkways of high human use in built environments with fragmented landscapes is as high, or higher than many endemic Hudson Valley counties [64] with the highest reported incidence of Lyme disease in New York State and among the highest in the nation [64,65]. Using the number of ticks tested and percent infected with *B. burgdSorferi* from data presented in Prusinski et al. [64], we calculated overall prevalence rate of infected ticks at 17.6% and 45.2% in nymphs and adults, respectively, among Hudson Valley counties, compared to infection rates of 27.5% and 45.5% for nymphs and adults, respectively, for the current study in Broome County (Table 1). All sites surveyed in this study are of high human use. The Binghamton University campus and the adjacent Nature Preserve are heavily used by faculty, staff, students, and the community. Wolfe Park and Chenango Valley State Park are heavily used by the community for recreational purposes. Many walkways have questing height vegetation growing onto the path or walkway, making it inevitable for walkway users to come in contact with vegetation, and thus, potentially with infected ticks.

Summer is considered the highest risk season for Lyme disease transmission [54,61], and health departments often stress that people should take appropriate precautions when entering forested environments [49]. However, it is worth noting that many cases diagnosed in the summer were transmitted in the spring, as symptoms do not usually occur for 3–30 days after a bite [40]. Our results show that although there is a higher density of ticks throughout the summer months (June through August) when smaller nymphal ticks are most active and less likely to be detected [48,49], tick infection and the density of infected ticks is proportionally much higher during spring (April and May) and fall (September to November). It is likely that public health precautions during summer months may cause people to infer that spring and fall pose less of a risk of infection. Yet, our data suggest that high tick density and infection rate in spring and fall months represent a significant population health risk (Table 1 and Table 2, Figure 3), a risk compounded by behavioral attitudes informed by the notion that built environments provide a safe haven from contact with potentially infected ticks. Seasonal variation in risk of transmission within built environments may also be influenced by local and regional climatic patterns associated with global climate change. Such influences have resulted in warmer wetter shorter winters in higher latitudes and earlier longer warmer seasons. These changes have contributed to the geographic expansion and growth of tick populations into more northerly areas of the US, Canada and Eurasia impacting areas not previously known for tick-bone disease [12].

Adult ticks have a higher infection rate than nymphal ticks, as they have two opportunities to take a blood meal, thus two opportunities to be infected with *B. burgdorferi* and it has been shown that transmission of infection to humans can occur in less than 24 h, with other tick-borne pathogens being transmitted in as few as 15 min [66] supporting the view that adult ticks in spring and fall months are a significant threat to human health [67,68,69]. Studies of seasonal variations in tick density and infection in Switzerland and Sweden show that adult ticks have a higher prevalence of infection than nymphal ticks, that density and infection are higher during spring [69], and that tick infection is reduced during summer months and early autumn [70], findings consistent with our results in this newly emerging Lyme endemic area in Upstate New York.

Although Lyme disease is the most common vector-borne disease in the Northeastern United States, other co-infections should not be overlooked. A study in New York State by Tokarz et al. [71] determined that 71% of all ticks tested harbored at least one tick-borne pathogen and our data indicate that precautions should be taken during all seasons in which ticks are active to avoid tick bites using permethrin treated clothing, DEET, IR3535, wearing light colored clothing with long sleeves and long pants, tucking pant legs into socks, doing frequent tick checks while outdoors, removing clothing immediately upon coming indoors and putting clothing through the dryer, as well as showering after outdoor activities [72,73,74]. 

## 5. Conclusions

We conclude that the high density of ticks infected with *B. burgdorferi* found in built environments with fragmented landscapes and high human activity presents an increased population risk of contact with infected ticks throughout all seasons in which ticks are active. Awareness of this increased risk within built environments will assist with health intervention and education programs directed at mitigating the increase in Lyme disease cases in human populations in current and emerging endemic areas. Future studies will include assessing multiple tick-borne pathogens to determine human risk in upstate New York.

## Figures and Tables

**Table 1:** Tick density and infection rate along 50 heavily traveled walkways by season, month and life cycle stage over a two-year period, from June 2012 through May 2014.

Season	Month	Total Ticks Collected		Total Area Dragged	Tick Density per 1000 m^2^		# Ticks Tested		% Ticks Infected	
		Nymphs	Adults	Square Meters	Nymphs (95% CI)	Adults (95% CI)	Nymphs	Adults	Nymphs (95% CI)	Adults (95% CI)
Summer 2012	June	Data not collected					15	3	46.7	66.7
	July						54	12	14.8	8.3
	Overall						69	15	21.7	20.0
Fall 2012	October	4	68	5304.4	0.8	12.8	4	68	50.0	47.1
	November	0	20	3051.6	0.0	6.6	0	18	n/a	83.3
	Overall	4	88	8356.0	0.5	10.5	4	86	50.0	54.7
Spring 2013	April	0	32	3172.1	0.0	10.1	0	29	n/a	37.9
	May	0	9	4829.0	0.0	1.9	0	9	n/a	33.3
	Overall	0	41	8001.1	0.0	5.1	0	38	n/a	36.8
Summer 2013	June	467	1	17,958.9	26.0	0.1	419	1	28.2	0.0
	Overall	467	1	17,958.9	26.0	0.1	419	1	28.2	0.0
Fall 2013	September	0	13	11,286.3	0.0	1.2	0	13	n/a	30.8
	October	10	525	53,048.4	0.2	9.9	10	521	30.0	38.2
	November	0	24	927.0	0.0	25.9	0	24	n/a	50.0
	Overall	10	562	65,261.7	0.2	8.6	10	558	30.0	38.5
Spring 2014	April	0	131	20,627.9	0.0	6.4	0	130	n/a	62.3 ^a^
	May	0	71	6407.1	0.0	11.1	0	65	n/a	72.3 ^a^
	Overall	0	202	27,035.0	0.0	7.5	0	195	n/a	65.6 ^a^
Summer 2012–Spring 2014		481	894	126,612.6	3.8 (±3.1)	7.1 (±3.1)	502	893	27.5 (±4.38)	45.5 (±2.83)
Overall		1375 *		126,612.6	10.9 (±2.19)		1395 *		39.0 (±2.38)	

^a^ Tick DNA was amplified using OspC primers and was 98% similar to LD1/LD2 primers; * Total ticks collected differ from total ticks tested so as not to alter density calculations because area dragged was not determined in Summer 2012.

**Table 2:** Density of infected ticks per 1000 m^2^ along heavily traveled walkways by season and life cycle stage based on 502 nymphal ticks and 893 adult ticks collected from fall 2012 to spring 2014. Overall figures were determined by calculating the sum of positive ticks and the sum of area dragged from all months and determining density of infected ticks per 1000 m^2^. ((Is the bold necessary?) (Is the capital necessary?)

Season	Month	Density of Infected Ticks	
		Nymphs	Adults
Fall 2012	October	0.4	6.6
	November	0.0	4.9
	Ovearll	0.2	5.5
Spring 2013	April	0.0	3.5
	May	0.0	0.6
	Overall	0.0	1.7
Summer 2013	June	6.6	0.0
	July	Data not collected for July and August. Cannot be calculated	
	August		
	Overall	6.6	0.0
Fall 2013	September	0.0	0.4
	October	0.1	3.8
	November	0.0	12.9
	Overall	0.1	3.3
Spring 2014	April	0.0	3.9
	May	0.0	7.3
	Overall	0.0	4.7
Fall 2012–Spring 2014		1.1 (±3.1)	3.2 (±3.1)
Total Ticks Fall 2012–Spring 2014		4.3 (±3.1)	
